# Assessment of Biosecurity Practices on Small Ruminant Farms in Kosovo After an Outbreak of Peste des Petits Ruminants: A Pilot Study

**DOI:** 10.3390/ani16121905

**Published:** 2026-06-19

**Authors:** Blerta Mehmedi, Shpetim Muharremi, Curtis R. Youngs, Imer Haziri, Arben Sinani, Hamdi Aliu, Gezim Hodolli, Sadik Heta, Armend Cana, Claude Saegerman

**Affiliations:** 1 Department of Veterinary Medicine, Faculty of Agriculture and Veterinary, University of Prishtina, 10000 Prishtina, Kosovo; blerta.mehmedi@uni-pr.edu (B.M.); shpetim.muharemi00@gmail.com (S.M.); imer.haziri@uni-pr.edu (I.H.); arben.sinani@uni-pr.edu (A.S.); hamdi.aliu@uni-pr.edu (H.A.); gezim.hodolli@uni-pr.edu (G.H.); 2 Animal Science Department, Iowa State University, 2356B Kildee Hall, Ames, IA 50011, USA; cryoungs@iastate.edu; 3 Kosovo Food and Veterinary Agency, Str. Lidhja e Pejes. No 241, 10000 Pristina, Kosovo; sadik.heta@rks-gov.net (S.H.); armend.cana@rks-gov.net (A.C.); 4 Microbiology Unit, University for Business and Technology-Higher Education Institution, Kalabria, 10000 Prishtina, Kosovo; 5 Research Unit in Epidemiology, Risk Analysis and Biosecurity Applied to Veterinary Sciences (UREAR-ULiege), Fundamental and Applied Research for Animals & Health (FARAH) Center, Faculty of Veterinary Medicine, University of Liege, 4000 Liege, Belgium

**Keywords:** sheep, goats, biosecurity, extensive, medium-scale farming, Kosovo, Peste des Petits Ruminants

## Abstract

Small ruminant production in Kosovo is predominantly extensive, and biosecurity practices remain poorly characterized. The emergence of Peste des Petits Ruminants (PPR) in Europe (beginning in 2024) and the first confirmed case in Kosovo (July 2025) highlight the urgent need for baseline biosecurity data to inform disease control. A cross-sectional pilot study was conducted on 63 small ruminant farms (53 meat-producing, 10 dairy-producing) across seven municipalities in Kosovo between September 2025 and February 2026. Biosecurity practices were assessed using the Biocheck.UGent™ questionnaire during direct on-farm visits. External (Ext) biosecurity scores (preventing pathogen introduction) were higher (*p* < 0.0001) than internal (Int) scores (limiting spread within farms). For external biosecurity, the highest scores were observed for purchase and reproduction (Ext A), intermediate scores existed for feed and water (Ext C) and visitors and farm workers (Ext D), and the lowest scores were found for transport and carcass removal (Ext B) and infrastructure (Ext E). For internal biosecurity, the highest scores were observed for lamb/kid management (Int H) and dairy management (Int I), followed by the management of adult animals (Int J); work organization (Int K) and reproduction management (Int G) formed an intermediate-low cluster, whereas disease management (Int F) scored the lowest. Benchmarking against the Biocheck.UGent™ worldwide database (predominantly intensive systems, thus not directly comparable) indicated that internal biosecurity and overall biosecurity levels were lower than the benchmark, while external biosecurity was comparable for some components. Given the convenience sample (36.4% response rate), findings are exploratory and are not directly generalizable. Larger herd size was positively correlated with external (ρ = 0.54, *p* < 0.0001), internal (ρ = 0.35, *p* = 0.005), and overall (ρ = 0.57, *p* < 0.0001) biosecurity scores. This first empirical biosecurity assessment of small ruminant farms in Kosovo reveals critical gaps in transport hygiene, disease management, and reproductive management pathways that enable PPR spread and perpetuate endemic zoonoses. The positive association between herd size and biosecurity may indicate structural barriers and/or knowledge gaps for small farms. Current biosecurity tools, designed for intensive systems, require adaptation for extensive production systems. These findings provide a baseline for targeted interventions, policy development, and validation of context-appropriate biosecurity instruments in Kosovo and similar extensive systems globally.

## Simple Summary

Small ruminant farming (sheep and goats) is very common in Kosovo, but little is known about how farmers protect their animals from infectious diseases. In 2025, an outbreak of Peste des Petits Ruminants, a highly contagious viral disease, reached Kosovo. This study assessed biosecurity practices on 63 farms using a standard questionnaire and direct farm visits. We found that farms do better at preventing diseases from coming in from the outside (for example, checking new animals and controlling visitors) than at stopping diseases from spreading within the farm. The weakest areas were transport hygiene, disease detection, and reproduction management. Larger farms had better biosecurity implementation. The tool we used was designed for large, intensive farms but does not fit well with Kosovo’s extensive management systems where animals graze together and move freely. Our findings show urgent gaps that need to be filled to control disease outbreaks and protect human and animal health. This study provides the first real-world picture of biosecurity in Kosovo’s small ruminant sector and points to the need for development of context-appropriate tools and practical support for farmers.

## 1. Introduction

The One Health paradigm recognizes the inextricable linkages between human, animal, and environmental health. Over 60% of emerging infectious diseases are zoonotic, and livestock production systems are major drivers of pathogen emergence [1]. Within this framework, biosecurity is defined as the integrated hygiene and management measures that reduce the probability of pathogen introduction and spread within and between livestock holdings [2]. Biosecurity operates across two complementary domains: external measures that prevent pathogen entry from outside the farm, and internal measures that limit spread once pathogens are present on a farm. Its effective implementation reduces zoonotic spillover, antimicrobial use, economic losses and environmental contamination, yet its application has been highly uneven across livestock sectors [3].

Small ruminants (sheep and goats) are a cornerstone of global agriculture, with a combined world population of over 2.4 billion animals [4]. They are critical for food security, household income, and social resilience in low- and middle-income countries [5]. Despite this importance, they have received substantially less scientific attention, policy prioritization and institutional investment than cattle, pigs or poultry. A workshop on brucellosis control explicitly noted that small ruminants “have been neglected in past research” [6]. A subsequent study described small flocks as “the missing link in control programmes”, demonstrating that they serve as unrecognized reservoirs representing “the starting point of new outbreaks at the livestock/human interface” [7].

The European Partnership on Animal Health and Welfare has identified biosecurity in small ruminant farms as a priority area for research [8]. A small set of high-leverage biosecurity controls for small ruminant farms has been identified: quarantine of purchased animals, segregation of risk groups, control of mixing points (e.g., communal grazing lands, markets), and traceability [9]. Implementation, however, remains poor across diverse production systems [10,11]. In low-resource settings, additional constraints, such as limited access to veterinary services and competing economic priorities, further impede implementation [12]. Smaller farm size and lower margins reduce the economic case for fixed biosecurity investments, as shown across livestock sectors [13].

Kosovo provides a relevant setting for examining and understanding these dynamics. Small ruminant production is widespread and predominantly based on extensive management with frequent use of shared grazing areas. A general livestock survey that included small ruminant farms reported that 81% of farmers lacked sufficient knowledge of biosecurity, indicating a major constraint to implementation [14]. This lack of knowledge is particularly concerning given recent disease events. Peste des Petits Ruminants (PPR), a highly contagious transboundary viral disease affecting small ruminants, emerged in Europe in 2024, with the first outbreaks reported in Greece and Romania (July 2024); these outbreaks were later characterized in a field study of affected sheep flocks [15]. Subsequent PPR outbreaks were reported in Bulgaria (November 2024) [16], Hungary (January 2025), Romania again (March 2025), Albania (June 2025), Kosovo (July 2025), and Croatia (December 2025) [17,18,19].

The first outbreak in Kosovo was confirmed on 1 July 2025, located on the border with Albania, where outbreaks had been confirmed weeks earlier [18]. Genomic analyses confirmed lineage IV in Greece, Romania, and Bulgaria [17]; spatial and temporal proximity suggest a common origin. As of April 2026, additional PPR outbreaks continued to be reported in Albania and Croatia, indicating that, despite control measures, the disease may still be circulating in the region [20].

Most biosecurity frameworks have been developed for intensive livestock systems, where animal contact can be limited, inputs monitored, and management practices standardized [21]. In contrast, extensively managed and medium-sized ruminant production systems, including those in Kosovo, operate under inherently limited control: animals share grazing areas, interact with other flocks, and move through informal trade networks. This creates a structural mismatch between conventional biosecurity assumptions and the realities of extensive and medium-sized systems.

The overall objective of the present study was to address this gap by providing an exploratory descriptive assessment of biosecurity practices in small ruminant farms in Kosovo using the Biocheck.UGent™ questionnaire [22,23]. This instrument was previously developed and validated for intensive pig and poultry systems but has not been validated for extensive small ruminant production. However, the tool has been used in Kosovo on dairy farms [24], which share some semi-intensive features. The specific objectives of this study were to: (1) describe the current level of external and internal biosecurity implementation; (2) identify the most frequently deficient biosecurity components; and (3) explore whether farm size is associated with total biosecurity score.

## 2. Materials and Methods

### 2.1. Study Design and Setting

**Figure 1:**
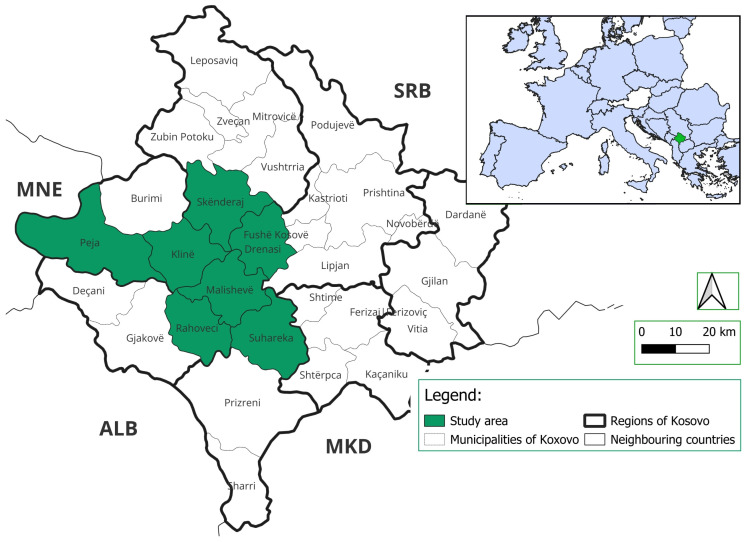
Map of the study area showing the seven municipalities (Pejë, Klinë, Skënderaj, Drenas, Malishevë, Rahovec, and Suharekë; shown in green) where small ruminant farms were sampled. Abbreviations for neighboring countries: MKD—North Macedonia; ALB—Albania; MNE—Montenegro; SRB—Serbia. The map is for illustrative purposes only; not to scale.

A descriptive, cross-sectional study was conducted to assess the implementation of biosecurity measures on a convenience sample of 63 sheep and goat farms (53 meat-producing, 10 dairy-producing) in Kosovo. The study was carried out between September 2025 and February 2026. Farms were contacted across 10 municipalities in Kosovo. However, farmers from only seven municipalities agreed to participate: Pejë, Klinë, Skenderaj, Drenas, Malishevë, Rahovec, and Suharekë (Figure 1).

### 2.2. Ethical Considerations and Informed Consent

The study was approved by the Subcommittee for Scientific Research of the Faculty of Agriculture and Veterinary Medicine, University of Prishtina (approval no. 2025/10). Before each interview, participants were informed about the study’s purpose, the voluntary nature of participation, and the confidentiality of their responses. All data were anonymized prior to analysis, and no identifiable farm information is reported. Data are stored on a password-protected computer accessible only to the research team. The assessor was directly supervised by the first author of this manuscript; no other conflicts of interest are declared.

### 2.3. Farm Selection

Farms for potential inclusion in this study were identified through local veterinary offices. Table 1 presents the number of farms contacted, the number that agreed to participate, and the participation rate for each municipality. Of 173 farms contacted, only 63 agreed to participate, yielding a response rate of 36.4%. Participating farms were located in seven municipalities (listed in Section 2.1). Five municipalities (Prizren, Dragash, Deçan, Lipjan and Ferizaj) had no participating farms.

Participating farms were selected using convenience sampling based on farmer willingness and the presence of active sheep or goat operations. No minimum flock size was imposed; the smallest farm had 10 animals, and the largest had 470 animals. All participating farms operated under extensive or semi-extensive management systems, characterized by seasonal mountain grazing and frequent inter-herd contacts during grazing or transport.

Given the exploratory nature of this pilot study and the absence of prior biosecurity assessments in Kosovo’s small ruminant sector, no formal power calculation was performed. The sample size of 63 farms reflects the practical reality of farmer willingness to participate. Despite contacting 173 farms across 10 municipalities, only 36% agreed to take part. This relatively low participation rate, while potentially introducing non-response bias, is itself informative about the challenges of conducting biosecurity research in this context; it is possible that farmers refused to participate due to absence of biosecurity measures or a myriad of other potential reasons. The achieved sample, though modest, is sufficient for an exploratory pilot study: it captures substantial variation in farm size (10–470 animals), includes both meat- and dairy-producing operations, and covers seven geographically diverse municipalities. This allows descriptive characterization of biosecurity practices, identification of major patterns and deficiencies, and generation of hypotheses for future larger-scale studies. Findings from this study are hypothesis-generating and not statistically powered for confirmatory inference.

### 2.4. Data Collection Instrument

Biosecurity practices were evaluated using the Biocheck.UGent™ questionnaire, a standardized tool developed by Ghent University. The original version of the questionnaire was used. No items were added, removed or reworded, and all questions were asked in full during every interview exactly as in the original instrument. This decision was made to maintain comparability with the existing Biocheck.UGent™ datasets, despite known limitations for extensive systems. The questionnaire is publicly available at https://biocheck.ugent.be.

### 2.5. Procedure

The assessor was a veterinary medicine student who completed one month of intensive training, which included a detailed review of each Biocheck.UGent™ questionnaire item and role-play interviews under the supervisor’s guidance. Pilot tests were conducted on the didactic farm of the Faculty of Agriculture and Veterinary Medicine, University of Prishtina. These pilots served to refine the conversational delivery and to practice cross-validation of verbal responses with direct observation. The data from the pilot farm were not included in the final analysis. For the 63 study farms, data were collected through on-farm visits by the same assessor and supervisor. The assessor and supervisor memorized the entire questionnaire before the start of data collection. This approach was chosen to avoid the use of visible electronic devices, which could make farmers nervous or inhibit natural conversation in the local context. During each visit, the interview was conducted in a relaxed conversational style while asking all questions in full, without omission or alteration of meaning. Concurrently, the assessor performed an observational walkthrough to visually assess farm infrastructure, hygiene practices, animal housing, grazing arrangements, and visible biosecurity measures (e.g., presence of quarantine pens, footbaths, visitor logs). On-farm interviews allowed the assessor to cross-validate verbal responses with direct observation. This approach follows a validation method [25], which demonstrated that on-farm visits can identify discrepancies between reported and actual biosecurity practices.

The assessor took short, structured handwritten notes during the interview, focusing on any discrepancies or clarifications. Immediately after each farm visit (within 2 h), the assessor transcribed responses onto the structured Biocheck.UGent™ form. Notes from the observational walkthrough were used to cross-validate verbal responses. This method prioritized accuracy over memorization; no responses were recorded from memory without contemporaneous notes.

When a discrepancy was identified between a farmer’s verbal response and the assessor’s direct observation, the final score was based on the assessor’s observation, following the principle that “what is observed supersedes what is reported” (i.e., based on clear evidence that contradicts reported practices). All discrepancies were documented in the field notes and reviewed with the supervisor during weekly debriefing sessions; no systematic pattern of discordance was identified across farms.

The supervisor accompanied the assessor on all farm visits (100%) to verify data consistency. During each visit, the supervisor independently completed a subset of key questionnaire items (covering all component areas) and compared findings with the assessor’s records. Agreement between the assessor and supervisor was consistently high (>90%) across all farms, with no systematic discrepancies observed. These data provide confidence in the reliability and consistency of the data collected.

The entire process (interview and walkthrough) took approximately 4 to 6 h per farm. No time constraints were imposed on the farmers. The extended on-farm duration was necessary to build rapport, allow farmers to recall routine practices without pressure, and verify responses across different farm areas (e.g., feeding areas, lambing pens, isolation facilities).

### 2.6. Variables Assessed

The Biocheck.UGent™ questionnaire yields scores for 11 specific components (A to K) subdivided into external and internal biosecurity measures (BSM):

External BSM: (A) purchase and reproduction (17 questions); (B) transport and carcass removal (9 questions); (C) feed and water (5 questions); (D) visitors and farm workers (13 questions); (E) infrastructure, location, and storage (10 questions).

Internal BSM: (F) disease management (10 questions); (G) reproduction management (13 questions); (H) lamb/kid management (8 questions); (I) dairy management (10 questions); (J) management of adult animals (4 questions); (K) working organization and equipment (7 questions). Note that (I) the dairy management component is applicable only for dairy small ruminants.

The Biocheck.UGent™ scoring algorithm followed a scientific, risk-based methodology developed by Ghent University. Instead of simple averages, the algorithm applied a weighted system formulated by international biosecurity experts that accounted for the relative importance and frequency of different pathogen transmission routes. The scoring process proceeded as follows:

Each closed question received a raw score based on the farmer’s response (typically 0 for ‘no’ or ‘never’, 1 for partial implementation, and 2 for ‘yes’ or ‘always’, though some questions used no/yes 0/1 binary scoring). Raw scores were transformed to a 0–100 scale using component-specific weighting factors derived from expert opinion and validated in previous studies. The official Biocheck.UGent™ weighting was applied without modification to maintain comparability with the existing database. A score of 0 indicated total absence of biosecurity measures for that component; a score of 100 indicated full implementation of all recommended measures.

For questions deemed “not applicable” to a particular farm (e.g., questions about artificial insemination for farms using only natural mating, or dairy-specific questions for meat farms), the scoring algorithm excluded those items and rescaled the remaining items within that component to a 0–100 basis. The assessor received training on the correct application of the ‘not applicable’ classification prior to data collection.

The dairy management component (Int I) consisted of 10 questions related to milking hygiene, milk storage, and udder health. It was administered only to the 10 farms classified as dairy-producing operations. For analyses requiring complete data across all farms (e.g., the overall comparison between external and internal scores presented in Section 3.2), the internal biosecurity score for meat farms was calculated as the average of components F, G, H, J, and K (excluding I). For dairy farms, the internal score included component I. This pragmatic approach allowed inclusion of all farms while acknowledging the structural difference between production types.

Total external, total internal, and overall biosecurity scores were calculated as the arithmetic means of the respective component scores.

Farm size was recorded as the total number of animals (sheep and goats combined). Production type was recorded as “meat” or “dairy”, based on the farmer’s primary product (milk vs. meat).

### 2.7. Data Analysis

#### 2.7.1. Effect of Herd Type

To determine whether data from meat-producing (n = 53) and dairy-producing (n = 10) farms could be pooled, we compared component scores between the two groups using the Wilcoxon signed-rank test and a Bonferroni correction. No differences were found. Given the exploratory nature and small dairy sample, data were pooled as a pragmatic decision.

#### 2.7.2. Normality Assessment

The Shapiro–Wilk test was applied to assess the normality of external, internal, and overall biosecurity scores.

#### 2.7.3. Comparison of Biosecurity Component Scores

Because component scores are repeated measurements within farms, the distribution of scores across the 11 components (A–K) was first compared using the extended Mantel–Haenszel (Cochran–Mantel–Haenszel) stratified test of association. For pairwise comparisons between specific external components (Ext A vs. Ext B, C, D, E) and between internal components (Int F, G, H, I, J, K), we used the Wilcoxon signed-rank test. Where only two components were compared (e.g., Int. J vs. Int. K, and external vs. internal overall scores), a Wilcoxon signed-rank test was applied. Bonferroni correction was applied within each comparison: 10 pairwise comparisons for external components (threshold *p* < 0.005), 15 for internal components (*p* < 0.00333), and 1 for the external-vs.-internal comparison (threshold *p* < 0.05).

#### 2.7.4. Herd Size Analysis

Species-specific analysis was not performed due to sample size constraints. Because the biosecurity scores were not normally distributed (Shapiro–Wilk test: external *p* = 0.02, internal *p* = 0.01, overall *p* = 0.01), Spearman’s rank correlation coefficient (ρ) was calculated to assess the relationship between herd size and each biosecurity score (external, internal, and overall). To visualize potential non-linear relationships, Locally Weighted Scatterplot Smoothing (LOWESS) was applied with a bandwidth of 0.8.

LOWESS is descriptive only; non-linearity was not statistically tested.

#### 2.7.5. Benchmarking

Component-specific scores were compared with current worldwide benchmark values available from the Biocheck.UGent™ public database (https://biocheckgent.com/). Differences were calculated as observed score minus benchmark value. No inferential statistics were applied to benchmarking comparisons; these are presented descriptively.

#### 2.7.6. Missing Data

No missing data were present for the 63 included farms, as the assessor completed all questionnaire items during on-farm visits. The dairy management component (I) was only applicable to the 10 dairy farms and was excluded from analyses requiring complete data across all farms.

Given the exploratory and pilot nature of this study, all *p*-values are reported descriptively and should be interpreted as hypothesis-generating rather than confirmatory. Findings from this study require validation in larger, hypothesis-driven studies.

## 3. Results

To provide an overview of the distribution of biosecurity scores across the 63 farms, descriptive statistics were calculated for each component of the Biocheck.UGent™ questionnaire. Table 2 presents the median, interquartile range (IQR), minimum, and maximum for all external and internal components, as well as for the aggregated external, internal, and overall biosecurity scores. The median is reported as a robust measure of central tendency due to the non-normal distribution of many components, and the IQR is used to describe the spread of the middle 50% of observations. The number of farms (n) is indicated for each component; note that the dairy management component (Int I) was assessed only on the 10 dairy farms.

### 3.1. Herd Type Effect on Components of Biosecurity

The effect of herd type (meat versus dairy) was tested on each component of biosecurity measured using the Wilcoxon signed-rank test and applying a Bonferroni correction. Because no effect of herd type was observed on all components of biosecurity, the data were further analyzed without separation between meat and dairy type, excluding benchmarking comparisons for which meat and dairy values were considered separately.

### 3.2. Component Biosecurity Scores

**Figure 2:**
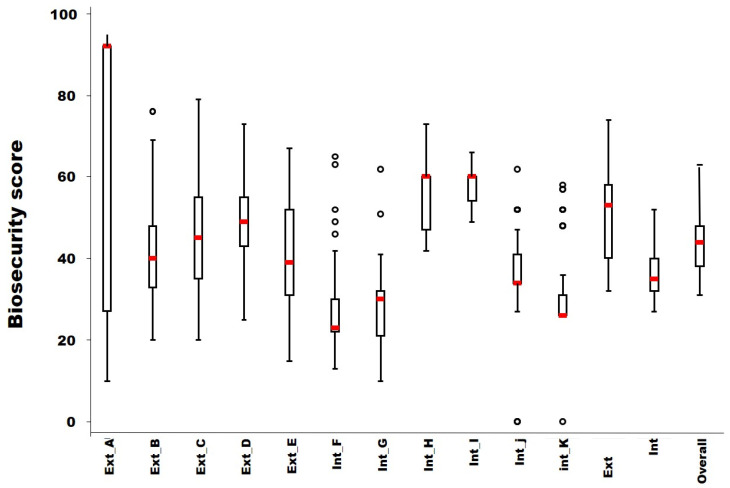
Boxplot of the score obtained for each component of external and internal biosecurity, as well as aggregate external, internal, and overall biosecurity. Legend: 95% confidence interval; small circles represent scores outside of the 95% confidence limit; the horizontal lines in red represent the median value of the biosecurity score. The solid lines and the top and bottom of each box represent the third and first quartiles, respectively.

The biosecurity scores for the individual components are presented in Figure 2. The scores were not equivalent across components (Extended Mantel–Haenszel stratified test; Q (13) = 344.58, *p* < 0.0001).

#### 3.2.1. External Biosecurity Components (n = 5 Components; 10 Pairwise Comparisons)

The score Ext (A) for “purchase and reproduction” was higher (Wilcoxon signed-rank test; *p* < 0.0001) than other external biosecurity components. When considering the other components of external biosecurity, Ext (D) “visitors and farm workers” and Ext (C) “feed and water” were similar to each other (Wilcoxon signed-rank test; *p* = 0.02) but were higher than Ext (B) “transport and carcass removal” and Ext (E) “Infrastructure, location, and storage” (Wilcoxon signed-rank test; *p* < 0.0001). No significant difference was found between Ext (B) and Ext (E) (Wilcoxon signed-rank test; *p* = 0.55).

#### 3.2.2. Internal Biosecurity Components (n = 6 Components; 15 Pairwise Comparisons)

The score Int (H) “lamb/kid management” and score Int (I) “dairy management” were similar (Wilcoxon signed-rank test; *p* = 0.96), and both were higher than other component scores (Wilcoxon signed-rank test; *p* < 0.0001). Excluding Int (H) and Int (I) in a subsequent analysis, the component Int (J) “management of adult animals” was higher than the remaining others (Wilcoxon signed-rank test; *p* ≤ 0.0024). In addition, the component score Int (K) “work organization and equipment” and Int (G) “reproduction management” were similar (Wilcoxon signed-rank test; *p* = 0.024), and both were higher than the Int (F) “disease management” (Wilcoxon signed-rank test; *p* ≤ 0.0028).

Additionally, the mean external biosecurity score was higher than the mean internal biosecurity score (Wilcoxon signed-rank test; *p* < 0.0001).

### 3.3. Herd Size Effect on Biosecurity Scores

**Figure 3:**
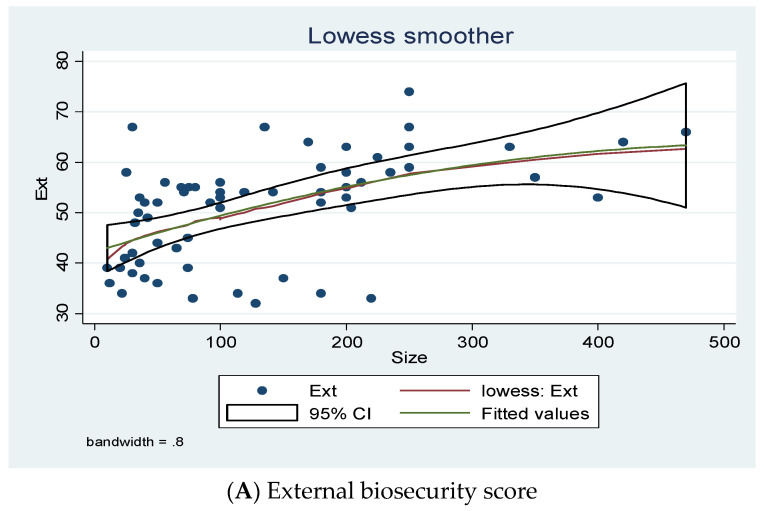

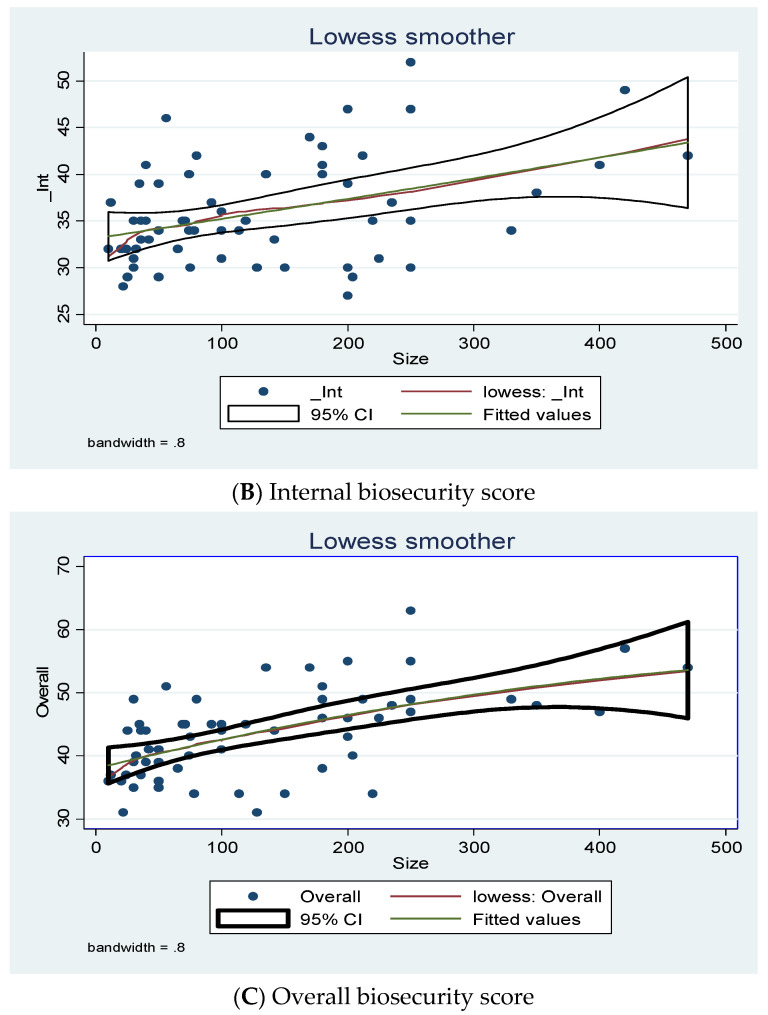
Relationship between the biosecurity score and herd size using the Locally Weighted Scatterplot Smoothing procedure (LOWESS smoother), applying a bandwidth of 0.8. Scores for each farm (dots) were plotted, values were fitted, and the 95% confidence interval is shown within the dark black lines. (**A**) external biosecurity score; (**B**) internal biosecurity score; (**C**) overall biosecurity score.

The minimum and maximum herd sizes were 10 and 470, respectively, with a mean of 132 (standard deviation: 108) and a median of 100 (interquartile range: 154). Because both external and internal biosecurity scores were not normally distributed (Shapiro test; *p* = 0.02 and *p* = 0.01, respectively), we calculated the Spearman rank correlation in order to assess the relationship between herd size and biosecurity scores. A non-parametric effect of herd size was observed with external, internal and overall scores of biosecurity (Figure 3). The Spearman correlation coefficient was 0.54 with *p* < 0.0001 for external score, 0.35 for internal score with *p* = 0.005, and 0.57 for overall score with *p* < 0.0001.

### 3.4. Descriptive Comparison with the Biocheck.UGent^TM^ Worldwide Database

**Figure 4:**
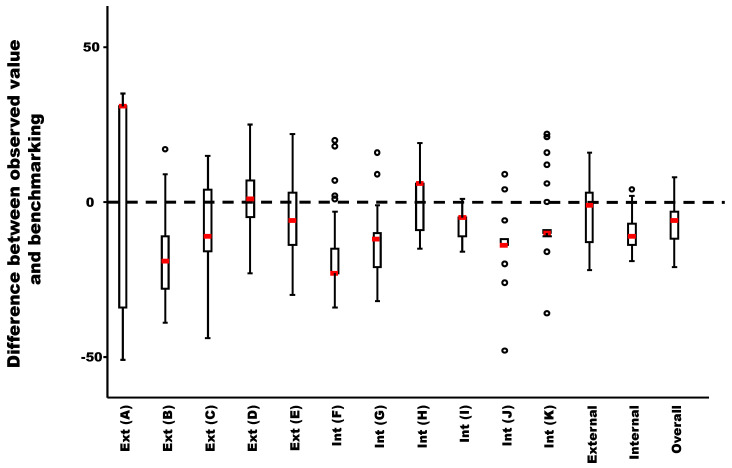
Difference between observed score of biosecurity by component and the current world benchmark value (presented for illustrative descriptive purposes only; not a normative standard). Legend: Ext: for external biosecurity; Int: for internal biosecurity; Overall: overall score of biosecurity; (A): purchase and reproduction; (B): transport and carcass removal; (C): feed and water; (D): visitors and farm workers; (E): infrastructure, location, and storage; (F): disease management; (G): reproduction management; (H): lamb/kid management; (I): dairy management; (J): management of adult animals; (K): work organization and equipment. The solid lines at the top and bottom of each box represent, respectively, the first and third quartiles; lines extending from the box, often known as whiskers, represent the limits of the 95% confidence interval. Small circles represent values lying outside the 95% confidence interval. The horizontal red lines represent the median value of the biosecurity score.

For illustrative purposes only, component-specific scores from this study were compared with current worldwide benchmark values available from the Biocheck.UGent^TM^ public database (https://biocheckgent.com/) (Table S1 and Figure 4). It is important to emphasize that this benchmark database is dominated by intensive production systems in high-income countries, and the Biocheck.UGent™ tool has not been validated for extensively managed small ruminant production systems. Therefore, these comparisons should not be interpreted as Kosovo farms ‘underperforming’ against a normative standard, but rather as highlighting structural differences between production system types. The observed differences likely reflect the inherent characteristics of extensive systems (e.g., shared grazing, limited infrastructure) rather than deficiencies per se.

With this caveat, we note descriptively that for external biosecurity the Ext (A) component “purchase and reproduction” and Ext (D) component “visitors and farm workers” showed values similar to the benchmark; while, Ext (B) “transport and carcass removal”, Ext (C) “feed and water” and Ext (E) “infrastructure, location, and storage” were lower. For internal biosecurity, except for Int (H) “lamb/kid management”, all components were lower than the current worldwide benchmark, indicating need for improvement.

## 4. Discussion

All statistical comparisons reported in this study are exploratory. No causal inference is attempted; associations are descriptive and hypothesis-generating. Findings should be interpreted with caution given the pilot nature of this work. Findings, nonetheless, will inform national policy, veterinary extension, and future research, including the need for development of a validated biosecurity assessment tool specifically for extensive management systems. To that end, given the absence of a validated tool for extensive systems, we used Biocheck.UGent™ exploratorily.

The principal finding of this pilot study was that external biosecurity scores were significantly higher than internal scores among 63 small ruminant farms in Kosovo. The higher scores for external measures may reflect that these align with routine trading and social practices (e.g., purchase inspection, visitor controls), whereas internal measures (e.g., disease surveillance, reproduction management) require sustained resources, training, and record-keeping. A similar trend of higher external scores was reported in Turkish small ruminant farms [9]. Conversely, Portuguese farms showed poor implementation across both domains, suggesting that standardized biosecurity plans may be ineffective without contextual adaptation [10]. In this regard, behavioral economic studies in the UK have shown that biosecurity adoption is influenced by perceived importance and knowledge, yet remains low despite net benefits [11]. Smaller farm size and lower profit margins also reduce the economic incentive for fixed biosecurity investments [13].

The highest external biosecurity scores were observed for Ext A “purchase and reproduction”, likely reflecting practices associated with routine livestock trading. Intermediate scores were observed for the Ext C “feed and water” and Ext D “visitors and farm workers”, which were statistically similar to each other. In contrast, the lowest external scores were found for Ext B “transport and carcass removal” and Ext E “infrastructure”. Among internal biosecurity components, the highest scores were for Int H “lamb/kid management” and Int I “dairy management”, followed by Int J “management of the adult animals at an intermediate level”. Int K “work organization and equipment” and Int G “reproduction management” were similar to each other and significantly higher than Int F “disease management”, which was the lowest-scoring internal component. This pattern suggests that farmers prioritize visible, production-oriented practices, while neglecting clinical surveillance and reproductive health management, with direct implications for both PPR spread and endemic zoonoses. Animal movements are a major pathway for transboundary disease transmission [26]. This risk is particularly pronounced for PPR, as a recent spatial risk mapping study across nine countries surrounding the Black Sea (including Bulgaria, Romania, and Turkey) also identified seasonal small ruminant movements as a major potential driver of spread [27]. As was found in the Romanian PPR characterization, animal movements were a key risk factor for the rapid spread of a highly virulent strain in large commercial flocks, a strain that circulated in a neighboring Balkan country just one year before the Kosovo outbreak [15]. On Kosovar farms, transport hygiene (Ext B), which directly controls animal movement risks, was among the lowest-scoring biosecurity components. The absence of vehicle cleaning and disinfection documented in Germany [28] creates a direct mechanism for PPR propagation. Modeling studies indicate that enhanced cleaning protocols can substantially reduce between-farm transmission risk [29]. In this study, no farm reported routine disinfection of transport vehicles. Carcass disposal is another critical gap; improper disposal can contaminate shared grazing lands and water sources. Pathogen survival in manure is well documented, and communal watering points (common in extensive systems) can harbor pathogens for weeks in manure-contaminated water [30]. The low score for “infrastructure” (Ext E) reflects the absence of dedicated quarantine facilities, a structural constraint also commonly observed elsewhere.

Larger herd size was positively correlated with biosecurity scores (external ρ = 0.54, internal ρ = 0.35, overall ρ = 0.57). This association is exploratory, however, because the cross-sectional design cannot distinguish whether larger herds invest more in biosecurity or whether biosecurity enables herd growth. The mechanism is likely multifactorial; larger herds generate greater revenues, thus enabling infrastructure investment, and they typically have more frequent veterinary contact. Reverse causality cannot be excluded. Psychosocial factors such as risk perception and social norms may influence biosecurity uptake even more strongly than farm size [31]. Even in high-income countries, biosecurity on sheep farms remains suboptimal, as shown in Finland [32], while Swedish farmers achieved higher internal biosecurity through strong veterinary extension systems [33].

The timing of this study, immediately following the first confirmed PPR case in Kosovo (July 2025), allows interpretation of biosecurity practices under real-world disease pressure. European PPR outbreaks in 2024–2025 were driven by livestock movements; index cases in Greece and Romania were traced to animal transport networks, with subsequent spread to Bulgaria, Albania, Kosovo, and Croatia along major routes [17,18,19]. The World Organisation for Animal Health issued alerts and recommendations [16,34]. The low score for “transport and carcass removal” (Ext B) is, therefore, particularly salient. The 2015 global strategy developed by FAO and WOAH to eradicate PPR by 2030, reaffirmed by a recent editorial [35], underscores the urgency of addressing biosecurity deficiencies such as those documented here. However, because data collection occurred after the outbreak, farmers’ awareness of PPR may have temporarily elevated biosecurity practices relative to pre-outbreak levels. Thus, the observed low scores indicate that, even under outbreak conditions, critical gaps remained.

Importantly, the PPR outbreak also highlights a potentially overlooked risk factor for extensive systems: wildlife contact. We emphasize that while our study did not systematically measure wildlife-livestock interactions, conceptual considerations suggest that outdoor farming may raise biosecurity concerns due to potential contact with infected wildlife. Previous research has shown that pathogen diversity can be higher on small ruminant farms compared to cattle or pig farms [36], and the wildlife-livestock interface can create opportunities for disease spillover and spillback [37]. In Kosovo production systems, these pathways could potentially contribute to disease introduction and persistence. However, because we did not systematically quantify wildlife contact frequency, duration, or species involved, nor document specific shared grazing arrangements, these mechanisms remain speculative. Future research should directly measure wildlife-livestock contact frequencies, characterize communal grazing networks, and assess their association with biosecurity outcomes and disease risk in extensive systems. Direct risk factor studies for the European PPR outbreaks are not yet available; evidence from other regions should be extrapolated with caution. Despite these well-established transmission mechanisms, empirical evidence on biosecurity implementation in extensive systems has been sparse. A recent cross-national survey across European small-scale farming systems confirmed that significant gaps remain, particularly in quarantine, hygiene, and visitor control practices [38]. Moreover, the combination of informal value chains and frequent wildlife contacts carries disproportionately higher risks of pathogen spillover [39].

In the present study, no farm had measures to mitigate wildlife contact, a critical blind spot in both the assessment tool and national biosecurity strategies [40]. Conventional biosecurity assumes dedicated quarantine facilities for newly purchased animals; in extensively managed and medium-sized systems, such infrastructure is often lacking. Likewise, standard protocols requiring visitor logs and protective clothing are seldom feasible, as visitors commonly move freely between farms without hygiene barriers. Communal grazing makes inter-flock contact difficult to prevent, and uncontrolled animal movements through informal trade networks bypass traceability systems. These tangible mismatches directly affect the feasibility and effectiveness of standard biosecurity measures. Future tools should incorporate these items identified here for use on extensive and medium-scale farms.

Low internal biosecurity scores for “disease management” (Int F) and “reproduction management” (Int G) may have consequences for human health. Small ruminants are reservoirs of zoonotic pathogens including *Brucella melitensis*, *Coxiella burnetii*, *Chlamydia abortus*, and *Toxoplasma gondii* [41]; *Brucella melitensis* from sheep and goats is the most common cause of human brucellosis worldwide [42]. Similarly, *Coxiella burnetii*, responsible for a Dutch Q fever outbreak with over 4000 human cases, remains under-recognized, with seroprevalence reaching 8.4% in sheep and 24.4% in goats in Spain [43,44]. Several infectious abortions in small ruminants are zoonotic (e.g., chlamydiosis, Q fever, campylobacteriosis, toxoplasmosis, and brucellosis), yet producer education remains inadequate [45].

The European Commission has long prioritized brucellosis control in sheep and goats [46]. In Kosovo, brucellosis remains endemic, with documented severe manifestations such as neurobrucellosis [47]. The present study suggests that Kosovar farms have similarly low internal control, yet farmer awareness of these zoonotic risks appears minimal [14]. The low scores for disease management also undermine early detection capacity; absence of systematic clinical records and isolation protocols means that index cases may go unrecognized, facilitating spread [48].

Benchmarking against the Biocheck.UGent™ worldwide database is presented for descriptive purposes only and should not be misconstrued as a normative standard for extensively managed and medium-sized ruminant production. The lower scores observed for most components in our study likely reflect structural differences in production models rather than the failure of Kosovar farmers. Nevertheless, the comparison exposes an important gap in the current evidence base, namely, extensive systems that are characterized by shared grazing, transhumance, and informal trade, which represent a substantial portion of the world’s small ruminant livestock across the Mediterranean, sub-Saharan Africa, Central Asia, and Latin America [49]. Livestock mobility remains a defining feature of these systems [50,51], yet no validated biosecurity assessment tool exists for such contexts. The gaps identified here in transport hygiene, disease management, reproduction and wildlife contact are, therefore, not anomalies but likely widespread across millions of farms, remaining severely under-documented. This study sparks an urgent call to the local, regional, and international research community: develop and validate fit-for-purpose biosecurity instruments for the world’s most prevalent yet most neglected livestock production systems. Failure to do so not only will perpetuate the vulnerabilities that enabled the 2025 PPR outbreak in Kosovo but also will continue to undermine One Health in the regions that need it most.

## 5. Limitations

This pilot study has several methodological limitations. The convenience sample and modest sample size prevent generalization to all Kosovar small ruminant farms; findings are exploratory. The relatively low farm participation rate (36.4%) introduces potential non-response bias. Farmers who agreed to participate may differ systematically from those who refused, for example, in awareness of biosecurity, herd size, or recent experience with disease. Consequently, the reported results could either overestimate or underestimate true implementation levels in the broader population. The timing of recruitment during the PPR outbreak also may have influenced participation.

The geographic scope of the study was limited to seven municipalities where farmers agreed to participate. Five municipalities had no participating farms. Results, therefore, may not represent other regions of Kosovo that have different production systems, disease pressures, or socio-economic conditions.

The Biocheck.UGent™ instrument has not been validated for small ruminant systems, limiting the applicability of its component scores in extensive production contexts. The tool was originally designed for intensive pig and poultry operations, and its assumptions (e.g., dedicated quarantine pens, controlled visitor access, record-keeping) do not fully align with the realities of extensive grazing systems, shared pastures, and informal trade networks. Consequently, the absolute score values should be interpreted with caution, and comparisons with intensive-system benchmarks are illustrative only.

Data were collected by a single assessor, and formal inter-rater reliability was not calculated across all farms. Additionally, no systematic data were collected on wildlife contact, communal grazing arrangements, vaccination history, farmer knowledge and attitudes, or economic constraints variables essential for understanding biosecurity drivers in extensive systems.

## 6. Conclusions

This exploratory pilot study provides the first empirical description of biosecurity practices on small ruminant farms in Kosovo following the 2025 PPR outbreak. The pattern of higher external than internal biosecurity measures indicates that farmers adopt practices aligned with routine trading, but neglect the clinical surveillance, record-keeping, and disease management needed to control outbreaks and zoonotic risks. The most severe deficiencies in transport hygiene, disease management, and reproduction are directly relevant to PPR spread and the persistence of endemic zoonoses such as brucellosis and Q fever. The results suggest that small- and medium-sized farms face structural barriers that cannot be overcome by awareness alone.

An important methodological finding is that current biosecurity assessment tools were designed for intensive, controlled systems and have limited applicability to extensive production systems characterized by shared grazing, seasonal mobility, and limited infrastructure. Key contextual factors such as wildlife contact and communal grazing arrangements are not captured by existing instruments. This is not a local anomaly; it is a global blind spot affecting extensive ruminant production systems across the Mediterranean, Africa, Asia, and Latin America.

Given the exploratory and pilot nature of this study (convenience sample, 36% response rate, seven municipalities), these findings should not be generalized to all Kosovar small ruminant farms. Instead, they serve three purposes: (1) providing a baseline for hypothesis generation; (2) identifying priorities for targeted intervention and policy development; and (3) demonstrating the need for validation of context-appropriate biosecurity instruments in extensive systems. Larger, representative studies with validated tools are needed to confirm and extend these findings, both in Kosovo and in similar production systems globally.

## Figures and Tables

**Table 1:** Farm recruitment and participation rates by municipality.

Municipality	Farms Contacted	Farms Participated	Participation Rate (%)
Pejë	21	21	100.0
Rahovec	15	13	86.7
Klinë	15	5	33.3
Malishevë	15	9	60.0
Skënderaj	10	4	40.0
Drenas	10	6	60.0
Suharekë	15	5	33.3
Prizren	21	0	0.0
Dragash	21	0	0.0
Deçan	10	0	0.0
Lipjan	10	0	0.0
Ferizaj	10	0	0.0
TOTAL	173	63	36.4

**Table 2:** Descriptive statistics for biosecurity component scores (0–100 scale) across 63 small ruminant farms in Kosovo.

Component	n	Median	IQR	Minimum	Maximum
EXTERNAL BIOSECURITY					
Ext A—Purchase and reproduction	63	92	65	10	95
Ext B—Transport and carcass removal	63	40	15	20	76
Ext C—Feed and water	63	45	20	20	79
Ext D—Visitors and farm workers	63	49	12	25	73
Ext E—Infrastructure, location, storage	63	39	19	15	67
INTERNAL BIOSECURITY					
Int F—Disease management	63	22	8	13	65
Int G—Reproduction management	63	30	11	10	62
Int H—Lamb/kid management	63	60	13	42	73
Int I—Dairy management	10	60	4.5	49	66
Int J—Management of adult animals	63	34	14	0	62
Int K—Work organization and equipment	63	26	10	0	58
AGGREGATE BIOSECURITY SCORES					
Total external (mean of A–E)	63	53	15	32	74
Total internal (mean of F–K, excluding I for meat farms)	63	34	8	27	52
Overall biosecurity (mean of external + internal)	63	44	8	31	63

Note: n = the number. IQR = interquartile range (Q3–Q1). Int I (dairy management) reported separately due to small sample (n = 10).

## Data Availability

Dataset available on request from the authors.

## Supplementary Materials

The following supporting information can be downloaded at: https://www.mdpi.com/article/10.3390/ani16121905/s1; Table S1: Difference between observed biosecurity score and the current world benchmark value, listed by biosecurity component.

## Abbreviations

The following abbreviations are used in this manuscript:

BSM	Biosecurity measures
DEFRA	Department for Environment, Food & Rural Affairs (UK)
EFSA	European Food Safety Authority
EU	European Union
EURL	European Union Reference Laboratory
Ext	External
FAO	Food and Agriculture Organization of the United Nations
Int	Internal
LOWESS	Locally Weighted Scatterplot Smoothing
PPR	Peste des Petits Ruminants
UK	United Kingdom
WOAH	World Organisation for Animal Health
