# Supplementary material for: Assessment of Biosecurity Practices on Small Ruminant Farms in Kosovo After an Outbreak of Peste des Petits Ruminants: A Pilot Study
Source: Animals (Basel). 2026 Jun 19;16(12):1905. doi: 10.3390/ani16121905 (PMC13296215; doi:10.3390/ani16121905)
Supplement: Supplementary file 1 [file animals-16-01905-s001.zip › animals-4366537-supplementary.pdf]

Table S1. Difference between observed biosecurity score and the current world benchmark value, listed by biosecurity component.

	Score obtained in this pilot study (N = 67)												Benchmark at world level		Difference between score and benchmark											
	Meat						Dairy						Meat	Dairy	Meat						Dairy					
Score	Min	Max	Mean	SD	Median	IQR	Min	Max	Mean	SD	Median	IQR	(N = 167)	(N = 113)	Min	Max	Mean	SD	Median	IQR	Min	Max	Mean	SD	Median	IQR
Ext (A)	10	93	66,28	32,44	92	65	11	95	63,30	33,49	92	69	61	60	-51	32	5,28	32,44	31	65	-49	35	3,30	33,48	32	69
Ext (B)	20	76	43,72	13,33	40	20	27	69	36,40	13,65	33	8	59	66	-39	17	-15,28	13,33	-19	20	-39	3	-29,60	14,42	-33	8
Ext (C)	27	66	44,38	10,09	40	20	20	79	49,60	11,47	54	8	51	64	-24	15	-6,62	10,09	-11	20	-44	15	-14,40	11,76	-10	8
Ext (D)	25	73	49,68	10,53	49	11	32	64	50,10	10,41	50	11	48	51	-23	25	1,68	10,53	1	11	-19	13	-0,90	10,47	-2	11
Ext (E)	15	67	39,42	13,60	39	19	35	52	45,60	12,80	52	16	45	49	-30	22	-5,58	13,60	-6	19	-14	3	-3,40	12,65	3	16
Int (F)	15	65	27,57	10,26	22	8	13	49	28,90	10,41	30	10	45	47	-30	20	-17,43	10,26	-23	8	-34	2	-18,10	10,42	-17	10
Int (G)	10	51	27,43	7,87	30	11	17	62	31,20	8,98	30	9	42	46	-32	9	-14,57	7,87	-12	11	-29	16	-14,80	8,87	-16	9
Int (H)	42	73	55,72	8,38	60	13	51	72	58,20	8,31	56	6	54	66	-12	19	1,72	8,38	6	13	-15	6	-7,80	8,95	-10	6
Int (I)	-	-	-	-	-	-	49	66	58,3	4,72	60	4,5	-	65	-	-	-	-	-	-	-16	1	-6,70	4,72	-5	5
Int (J)	0	52	36,49	11,89	34	0	27	62	36,50	12,02	33	14	48	53	-48	4	-11,51	11,89	-14	0	-26	9	-16,50	12,17	-20	14
Int (K)	0	58	30,87	11,61	26	0	26	48	35,30	11,77	29	22	36	42	-36	22	-5,13	11,61	-10	0	-16	6	-6,70	11,69	-14	22
External	32	67	50,62	10,21	53	15	36	74	50,40	10,81	55	20	53	58	-21	14	-2,38	10,21	0	15	-22	16	-7,60	10,98	-4	20
Internal	27	49	35,09	5,26	34	8	34	52	40,50	5,68	41	5	45	53	-18	4	-9,91	5,26	-11	8	-19	-1	-12,50	5,42	-13	5
Overall	31	57	43,13	6,49	44	8	35	63	45,60	6,94	47	10	49	56	-18	8	-5,87	6,49	-5	8	-21	7	-10,40	7,09	-9	10
